# The Role of Epigenetic Switches in Polyphenism Control: Implications from a Nematode Model for the Developmental Regulation of Alternative Phenotypes

**DOI:** 10.3390/biology13110922

**Published:** 2024-11-13

**Authors:** Sara Wighard, Ralf J. Sommer

**Affiliations:** 1 Max Planck Institute for Biology Tuebingen, Department for Integrative Evolutionary Biology, 72076 Tuebingen, Germany; sara.wighard@tuebingen.mpg.de; 2 Institute of Molecular Biotechnology of the Austrian Academy of Sciences (IMBA), Vienna BioCenter (VBC), 1030 Vienna, Austria

**Keywords:** developmental plasticity, *Pristionchus pacificus*, *eud-1*/sulfatase, *Arabidopsis*, *flowering locus C*, hymenopterans, stochasticity

## Abstract

Polyphenisms, the capability of organisms to form two or more alternative phenotypes in response to environmental variation, are prevalent in nature. However, associated molecular mechanisms and potential general principles of polyphenisms among major organismal groups remain currently unknown. This review focuses on an emerging model system for developmental plasticity and polyphenism research, the nematode *Pristionchus pacificus* and explores mechanistic insight obtained through unbiased genetic, experimental and natural variation studies. Resulting findings identify a central role for epigenetic switches in the environmental control of alternative phenotypes and their micro–and macroevolution. Several features observed in *P. pacificus* are shared with insects and plants and might become general principles for the control of polyphenisms during development.

## Simple Summary

Polyphenisms, the most extreme form of phenotypic plasticity, translate continuous environmental variation into discrete phenotypes in both animals and plants. While deeply embedded in the development of the individual organism, the mode of developmental regulation of polyphenisms has not been intensively studied for decades. Similarly, few attempts were made to search for conceptual similarities of polyphenisms across organismal groups. This situation is currently changing with the establishment of new model systems and advanced technological platforms. Here, we review mouth-form polyphenism in the nematode *Pristionchus pacificus* and compare associated molecular mechanisms to polyphenisms in insects and plants. These comparisons identify epigenetic switches as central features in the regulation of polyphenisms, with conserved system properties found in diverse organisms, including nematodes, insects, plants and human cancer progression. Thus, polyphenisms might provide important insight into the Nature vs. Nurture debate and help towards a better integration of genetic and environmental influences in health and disease.

## 1. Introduction

One of the universal properties of multicellular organisms is their environmental responsiveness, resulting in morphological, physiological, and behavioural variation that can be independent of the genotype [1,2,3]. Such phenotypic plasticity can occur in many forms and is often continuous. However, the most extreme types of phenotypic plasticity are those that result in alternative, discontinuous phenotypes, known as polyphenisms. The nonlinear relationship between environmental variation and organismal readout in examples of polyphenisms has been predicted to be of evolutionary significance [3], a hypothesis that is eventually gaining strong support after a long phase of criticism and debate (for recent reviews, see [4,5]). Additionally, the discrete nature of this phenotypic variation makes polyphenisms an ideal study system to investigate underlying molecular mechanisms. Insects are the champions of polyphenisms [6], with many examples from butterfly wing patterning [7], beetle horn development [8], locust stationary vs. migratory morphs [9] to aphids, with their different life cycles and reproductive strategies [10]. The most striking examples of polyphenisms, however, are those resulting in insect societies found in termites and all major groups of hymenopterans. In addition to the separation of queens and workers among female individuals, many ant or termite species have several discrete worker castes, resulting in complex morphological patterns in individual societies [11,12]. Some of the citations above represent exhaustive monographs that can give the interested reader a detailed overview of individual examples of polyphenisms.

All morphological polyphenisms have a developmental basis and are the result of reprogramming during individual development. Surprisingly, however, the underlying developmental processes have not been subjected to detailed investigations for many decades. This was due to two major reasons. First, insect societies have primarily been studied in an evolutionary context, with a strong focus on kin selection [13]. In contrast, developmental aspects, particularly the developmental plasticity of hymenopteran caste formation, were mostly side-lined and ignored (for critical review, see [14]). Second, developmental biologists have not paid much attention to polyphenisms, partly because no model systems with sophisticated toolkits have been available for many years. However, this situation has been changing in the last decade, with research in multiple study systems identifying regulatory mechanisms of developmental plasticity, particularly polyphenisms. Besides insects, amphibians and nematodes have emerged as promising study systems that allow molecular developmental processes to not only be identified but also linked to the ecological and evolutionary significance of polyphenisms [5]. Here, we review studies on the nematode *Pristionchus pacificus*, an emerging model system of plasticity and polyphenisms, and compare associated molecular principles with findings from insects and plants.

## 2. Evolutionary Transitions from Continuous Plasticity to Polyphenisms 

Polyphenisms require developmental reprogramming and are thought to have evolved from continuous plasticity through the introduction of thresholds between phenotypes. Pioneering work by Mary Jane West-Eberhard, Fred Nijhout, and Paul Brakefield on wasps and butterflies laid the foundation for polyphenisms as a developmental phenomenon with important evolutionary implications. The comparison of reproductive and non-reproductive individuals in solitary and social wasps resulted in the ovarian ground plan hypothesis dating back to the 1970s [15]. This hypothesis and its following modifications helped to understand two important features of polyphenisms in wasps [16]. First, cycles of different ovarian activity and context-dependent (environmental) expression of alternative behaviours result in the dichotomy between workers and queens. Consequently, caste differentiation requires developmental reprogramming. Second, a switch-like mechanism must exist that controls the development of different phenotypes and reprogramming towards alternative developmental pathways without intermediate phenotypes. These studies profited from their comparative and evolutionary perspectives and were further developed in butterflies. Evolutionary transitions of wing spot patterns in *Bicyclus* butterflies strongly supported the fundamental role of thresholds in the generation of alternative phenotypes [17]. Indeed, a broader comparison of butterfly wing patterns identified polyphenisms as threshold traits and provided evidence for the adaptive value of polyphenisms [18]. However, the molecular nature of the required developmental switches and associated threshold effects remained elusive. Work in nematodes has revealed the earliest insights into the molecular underpinnings of polyphenisms. For instance, the dauer vs. non-dauer decision in *Caenorhabditis elegans* involves the nuclear-hormone-receptor (NHR) DAF-12 and the Forkhead-type transcription factor DAF-16 as part of complex gene regulatory networks (GRNs) [19]. Below, we will introduce *P. pacificus*, which exhibits another type of nematode polyphenism with morphological and behavioural implications that are unknown from *C. elegans*.

## 3. *Pristionchus* Nematodes as Genetic Models for Polyphenism Research

**Figure 1:**
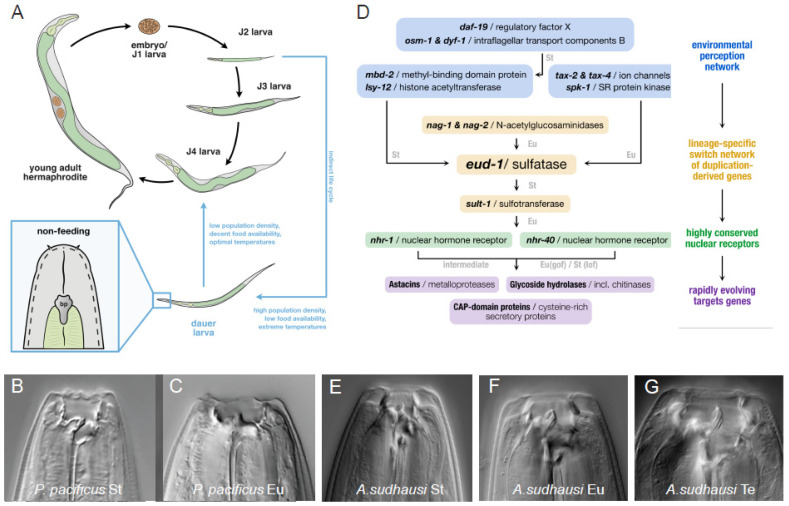
Nematode biology and mouth-form polyphenism. (**A**) Life cycle of *Pristionchus pacificus*. This nematode has four juvenile stages (J1–J4) with the J1 stage remaining in the eggshell so that the J2 stage hatches from the egg. Under favourable conditions, such as in the laboratory, the life cycle is completed within 4 days (20 °C). Under unfavourable conditions, worms enter an arrested, stress-resistant dauer stage, in which animals do not feed. (**B**) The stenostomatous (St) non-predatory mouth form of *P. pacificus* with a single tooth. (**C**) The eurystomatous (Eu) predatory mouth form of *P. pacificus* with two teeth and a broad buccal cavity. (**D**) Gene regulatory network of *P. pacificus* mouth-form polyphenism. Eu and St indicate the phenotype of respective null mutants. See text for details. (**E**–**G**) The distantly related nematode *Allodiplogaster sudhausi* forms three distinct morphs. Besides the St (**E**) and Eu (**F**) morph, they form a large-mouthed morph termed ‘teratostomatous’ (Te) (**G**), which is cannibalistic against kin. Note that the microscopy images of the two species are not similar in scale. The diameter of the worm at the tip of the adult head is appr. 30 µm in *P. pacificus* and 50 µm in *A. sudhausi*.

The nematode *P. pacificus* was first established as a satellite organism for comparison with *C. elegans* and shares a similar life cycle (Figure 1A) [20,21]. Both of these nematodes have several important technical features in common that allow genetic and molecular investigations. For example, *P. pacificus* has a four-day generation time (20 °C) in the laboratory, can be fed with *Escherichia coli* on agar plates, and is a self-fertilizing hermaphrodite, resulting in isogenic cultures. Forward genetic mutagenesis, reverse genetic engineering using CRISPR, and multiple -*omics* platforms are available and regularly updated [22,23,24,25,26]. In addition, studies in *P. pacificus* profit from a micro- and macro-evolutionary perspective and the availability of many nematode isolates and species as living cultures, i.e., more than 1500 *P. pacificus* wild isolates [27], around 50 *Pristionchus* species [28,29], and more than 30 genera of Diplogastridae [30], the family to which *P. pacificus* belongs. Note that in the absence of a detailed fossil record, the evolutionary divergence between nematode lineages has been the subject of a long-standing debate, with the last common ancestor of *P. pacificus* and *C. elegans* speculated to have lived between 100–400 MYA. However, recent studies involving molecular and geological parameters have begun to provide more accurate assumptions and suggest a split between 200–250 MYA [31,32].

*Pristionchus* and other diplogastrid nematodes exhibit a feeding polyphenism with two alternative phenotypes that is unknown from most other nematodes (Figure 1B,C). This feeding polyphenism represents an evolutionary novelty and is an example of functional diversification with strong ecological consequences [33]. During post-embryonic development, *Pristionchus* nematodes form teeth-like denticles in their mouth that culminate in the formation of discrete morphs in the adult stage, termed eurystomatous (Eu) and stenostomatous (St) [34,35]. St animals have a single flint-like dorsal tooth, a narrow mouth and are strict bacterial feeders (Figure 1B). In contrast, Eu animals have two teeth, one dorsal and one sub-ventral; a broader mouth, and can both feed on bacteria and predate on other nematodes (Figure 1C). Thus, this morphological polyphenism has important behavioural implications, similar to the distinction of female solitary wasps that turn into reproductive or non-reproductive individuals. 

The *P. pacificus* feeding polyphenism exhibits an interesting element of stochasticity. Specifically, the genetically identical progeny of a single hermaphrodite will form both alternative morphs at strain-specific ratios of Eu:St animals, even when grown under constant environmental conditions in the laboratory [36,37]. This stochasticity points towards the existence of a bistable switch, similar to the lysogenic switch of bacteriophage lambda and examples of phenotypic heterogeneity in various bacteria [38]. While it is outside the focus of this review, this mouth-form polyphenism permits potential cannibalism and surplus killing, which has resulted in a self-recognition system that affects collective behaviour [39,40,41,42]. Importantly, the formation of two alternative mouth forms and predation have to be seen in their ecological context. Many members of the Diplogastridae are found in association with insects [43], with the majority of *Pristionchus* species being isolated from scarab beetles [44]. For example, *P. pacificus* is often found on the Oriental beetle *Exomala orientalis* in Japan as well as North America after the invasion of the beetle [45]. Interestingly, in contrast to many other *Pristionchus* species, *P. pacificus* shows an atypically broad host range that facilitated the invasion of many continents and islands [27,46]. The *Pristionchus*-beetle association has been studied in great detail but is outside of the focus of this review [47,48,49]. Similarly, the evolutionary diversification of feeding structure morphologies in the context of plasticity, which strongly supports the adaptive value of this polyphenism, is outside of the focus of this review and we refer interested readers to other texts [30,50].

## 4. A Developmental Switch Regulates Mouth-Form Plasticity in *Pristionchus*

Like other developmental processes, the regulation of polyphenisms can best be investigated using genetic approaches. *P. pacificus* mouth-form polyphenism has been subjected to both unbiased forward and reverse genetic analyses over the past decade. Surprisingly, the first forward genetic screen for mouth-form defective mutants identified dominant mutations that act in a dosage-dependent manner and completely eliminate the Eu morph. The corresponding gene, termed *eud-1* for eurystomatous-form defective, encodes a sulfatase and is part of a developmental switch [51]. Heterozygous *eud-1*/+ animals have an Eu-defective phenotype and over-expression of a wild-type copy of *eud-1* rescues the *eud-1* mutant phenotype. Later experiments revealed that a large deletion of the *eud-1* locus has a phenotype similar to mutations resulting in single amino acid substitutions [52], which together indicate that the dominant *eud-1* phenotype results from *reduction*-or *loss-of-function* rather than *gain-of-function* mutations. When *eud-1* was over-expressed in *P. pacificus*, transgenic animals from wild isolates that are preferentially St instead adopted the Eu mouth form. This was even seen in the sister species *P. exspectatus*, which normally also exhibits the St morph. Overall, these findings support the notion that the EUD-1/sulfatase is part of a developmental switch and that the *P. pacificus* mouth-form polyphenism is a threshold trait [51].

Subsequent studies indicated that the *eud-1* locus, and the associated gene regulatory network (GRN), is extremely complex and contains many genes with surprisingly different evolutionary histories (Figure 1D). For example, the *eud-1* locus contains an anti-sense message on the opposite strand [53] and is part of a supergene locus with two more enzyme-encoding genes (*nag-1* and *nag-2*, encoding n-acteyl-glucosaminidases), which also regulate the feeding polyphenism (Figure 1D) [52]. Several *eud-1* suppressor screens identified two nuclear hormone receptors, *nhr-40* and *nhr-1*, that act downstream of *eud-1* and the sulfotransferase *seud-1/sult-1*, which functions downstream or in parallel [54,55,56,57]. For *nhr-40*, *gain-of-function* and *loss-of-function* alleles are available, which have an all-Eu and all-St phenotype, respectively, further highlighting their role in developmental switching [57]. Various other regulators were identified in these screens, including a gene encoding the Mediator subunit MDT-15.1 [58,59]. Together, the successful use of developmental genetic approaches resulted in an unprecedented understanding of a GRN controlling a polyphenic animal trait (Figure 1D). It is important to note, however, that our knowledge of the GRN is still far from being complete. In particular, in the current GRN, the targets of NHR-1 and NHR-40 largely encode for enzymes, i.e., metalloproteases and chitinases, that likely remodel the teeth. In contrast, we have not yet identified genes encoding for proteins that form the teeth and other differential structures of the mouth, which hopefully will be identified through future unbiased genetic and/or experimental approaches.

Nematodes are known for their huge genomic diversity and the rapid turnover of a large fraction of genes in their genomes [60,61]. Notably, gene turnover had a large impact on the composition of the *P. pacificus* mouth-form polyphenism GRN. Essentially, the GRN consists of three modules with different functions and genes of distinct evolutionary histories (Figure 1D) [62]. First, an upstream ‘environment-perception module’, containing the highly conserved ion channels *tax-2* and *tax-4* and cGMP signalling involving *daf-11*, is involved in the direct perception of environmental signals [63]. These genes are one-to-one orthologous to genes in *C. elegans* and other nematodes and share a function in environmental perception, arguing for the co-option of ‘old genes’ during the acquisition of a GRN controlling a new polyphenism. Second, the ‘plasticity switch module’ contains the genes *eud-1*, *nag-1*, *nag-2*, and *seud-1/sult-1*, as discussed above. Interestingly, these genes, with their crucial role in developmental switching, evolved through recent gene duplication events and have no one-to-one orthologs in *C. elegans* (Figure 1D) [52]. Finally, the ‘phenotype-execution module’ consists of the highly conserved transcription factors *nhr-1* and *nhr-40* and their rapidly evolving target genes, which include astacin/metalloproteases and chitinases, among others [57]. The integration of rapidly evolving genes into ancestral gene networks was further supported by co-expression analysis, highlighting the complex evolutionary history and dynamics of GRN composition, which likely holds true beyond nematodes [64]. The unexpected complexity of the evolutionary history of this GRN is subject to ongoing investigations, similar to studies that aim to identify additional regulators of the phenotype-execution module [65,66].

## 5. GRNs Controlling Polyphenisms Share General Principles

The detailed characterisation of the GRN controlling mouth-form polyphenism has three important implications. First, it testifies that plastic trait formation relies on genetic programs with principles similar to hard-wired and robust developmental processes. This finding should help towards a broader acceptance of polyphenisms and phenotypic plasticity in general, as an important principle in developmental *and* evolutionary biology. Second, knowledge about the composition of this network opens up opportunities to identify the mechanisms by which the polyphenism decision is reached. One likely possibility is that transcriptional switches allow alternative readouts, although the exact molecular mechanisms have yet to be identified. However, the fact that a sulfatase (*eud-1*), a sulfotransferase (*seud-1*/*sult-1*), and an NHR (*nhr-40*) are part of the developmental switch suggests a key role in transcriptional regulation. 

Finally, the emerging regulatory patterns of the mouth-form polyphenism are in strong agreement with findings in insects. For example, wing polyphenism in pea aphids is controlled by ecdysone signalling, the most prominent NHR signalling in insects [67]. Strikingly, regulators of aphid wing polyphenism also exhibit a diversity of evolutionary histories and even include a gene acquired by horizontal gene transfer from a virus [68]. In plant hoppers, two insulin receptors determine another type of alternative wing polyphenism [69], and insulin signalling was also shown to control eusociality in ants [70]. Finally, the transcription factor *doublesex* controls the plasticity of beetle horn development [71] and worker-associated traits in honeybees [72,73]. Thus, polyphenisms are often regulated by conserved transcription factors and involve GRNs that contain recently evolved or acquired genes. Thus, switching of transcriptional activity is one likely mechanism for the control of polyphenisms. 

## 6. The *Pristionchus* Mouth-Form Decision Turns into an Epigenetic Switch

**Figure 2:**
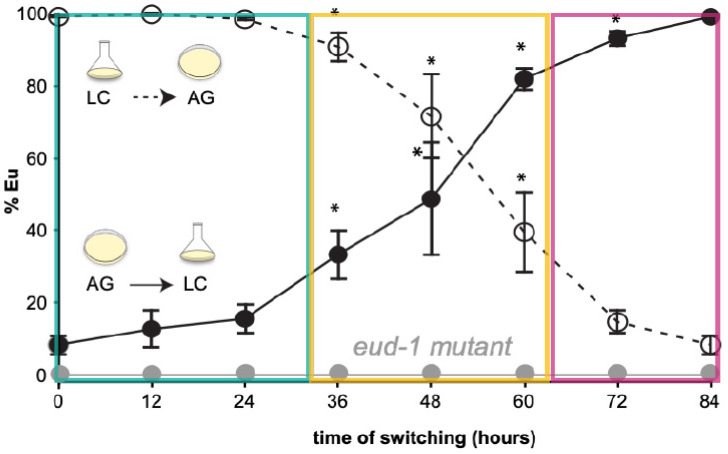
Culture switch experiments delineate developmental phases of polyphenism. Y-axis depicts that Eu mouth-form phenotype in percent. *P. pacificus* cultures on agar plates (AG) are largely Eu, whereas cultures in liquid (LC) are preferentially St. In this experiment, culture conditions of synchronised worm cultures at respective time points (x-axis) were switched. The graphs depict adult phenotypes and are plotted as a function of transferring between environments after synchronisation. When worms were transferred early in juvenile development (0–24 h), they showed no memory and expressed the phenotype of the new culture environment (marked in green). In contrast, if they were transferred late in juvenile development (beyond 60 h), they expressed the mouth-form phenotype of the first condition (marked in magenta). Surprisingly, there is a long window (36–60 h) around the third juvenile (J3) stage with intermediate ratios, that is, some animals show memory and others do not (marked in yellow). This indicates an epigenetic mechanism. Note that mutants of the *eud-1*/sulfatase are always all-St, regardless of culture conditions and switching. Statistical significance was assessed by binomial logistic regression on Eu and St counts between a given time-point, and the t’ = 0- and 84-h phenotype; *p*-values were adjusted by Bonferroni correction, * *p* < 0.025 relative to both. Error bars represent S.E.M. for n = 5 independent worm populations. Redrawn with permission from Werner et al., *Nature Communications* 2023, *14*, 2095.

Developmental switches are not restricted to polyphenisms, but their environmental responsiveness and binary (nonlinear) readout require additional network properties [74]. Several studies indicate the *P. pacificus* mouth-form switch is indeed an epigenetic phenomenon. Early studies already showed that culturing *P. pacificus* wild-type PS312 animals on solid agar plates with *E. coli* as a food source, results in preferentially Eu cultures, whereas liquid cultures of the same strain and diet are largely St [75]. This unexpected finding introduced a simple, but extremely powerful tool for experimental manipulation. Indeed, reciprocal transplant experiments between agar plate and liquid culture conditions at different times during postembryonic development revealed an unexpectedly long window of environmental sensitivity of mouth-form execution (Figure 2). This finding enabled the use of chemical inhibitor studies to identify associated molecular mechanisms. Trichostatin A, a known inhibitor of histone deacetylases (HDAC), was found to block the formation of St animals in liquid culture. Coupling reciprocal transplant experiments with the use of trichostatin A, and associated biochemical studies, led to the identification of acetylation at histone 4 lysine 12 (H4K12) and H4K5 at the *eud-1* locus in controlling the environmental responsiveness of mouth-form plasticity [35]. Thus, the mouth-form developmental switch gene *eud-1* is under the influence of epigenetic control, allowing the adjustment of a morphological and behavioural trait to specific environmental conditions. The same study also linked H4K12ac to controlling developmental speed, a function that was shown to be conserved even in *D. melanogaster* [35]. Taken together, the developmental switch controlling mouth-form polyphenism can be influenced by histone modifications, transforming the binary mouth-form decision into an epigenetic switch phenomenon.

The control of mouth-form polyphenism through histone modifications has greater complexity, as indicated by a recent study that points towards the role of the histone demethylase gene *spr-5* [76]. This gene has been identified through phylogenetic, transcriptomic, and bioinformatic analyses among various *P. pacificus* strains, as well as *Pristionchus* and other diplogastrid species. When CRISPR-induced *spr-5* mutants were generated in *P. pacificus*, the authors indeed found a mouth-form phenotype [76]. However, it remains currently unknown whether *Ppa*-SPR-5 also affects histone modifications at the *eud-1* locus. This complex mediation of feeding polyphenisms through epigenetic processes is exciting but also represents a challenge for future studies as epigenetic processes usually evolve rapidly [77]. For example, a recent catalogue of the *P. pacificus* epigenetic toolkit revealed the unexpected loss of the histone methyltransferase complex PRC2 within the genus *Pristionchus* [78]. This finding is surprising for two reasons. First, while the PRC2 complex is absent in *P. pacificus*, the PRC2-regulated histone mark H3K27me3 is still present, arguing for another writer to set this modification. Second, not all *Pristionchus* species have lost the PRC2 complex, suggesting that large-scale epigenetic alterations can occur between closely related species without any obvious sign of associated morphological or physiological consequences. As a result of these findings, candidate gene approaches are unlikely to identify the histone modifiers regulating feeding polyphenism; instead, systemic approaches that target the epigenomic toolkit will be necessary.

## 7. Epigenetic Reprogramming Underlies the Control of Polyphenisms

Regulating polyphenisms through epigenetic reprogramming, as indicated above for *P. pacificus*, allows a fine-tuned response to environmental variation. Indeed, a larger body of evidence suggests that the regulation of polyphenisms is often associated with epigenetic mechanisms and developmental reprogramming. A landmark paper in 2016 described the epigenetic reprogramming of caste-specific behaviour in the carpenter ant *Camponotus floridanus* [79]. These ants produce two distinct worker classes, so-called minors and majors, with the former showing stronger foraging and scouting behaviours. While earlier work by the same authors had already suggested a role of histone acetylation [80], they found the use of HDAC inhibitors enhanced the foraging behaviour among worker individuals, whereas histone acetyltransferase inhibitors could suppress this enhancement [79]. HDAC inhibitors not only changed the behaviour of workers towards the minor caste fate but transcriptome sequencing and chromatin analysis in the brains of treated animals strongly supported the role of H3K27ac in the developmental regulation of this caste polyphenism. Note that H3K27ac is a ubiquitous histone modification involved in many regulatory processes throughout animals and plants, whereas the H4K12ac mark identified in *P. pacificus* mouth-form polyphenism is rare. 

While the GRN controlling caste differentiation in carpenter ants is not fully understood, the regulation through H3K27ac is intriguing. However, the carpenter ant story is more complex because, similar to other hymenopterans, they additionally use DNA methylation for epigenetic programming [81]. The existence of histone modifications and parallel DNA methylation allows for the interplay of two major epigenetic mechanisms in the regulation of polyphenisms in hymenopterans and other insects, whereas DNA methylation is absent in *P. pacificus* and most other nematodes. Note that, however, the epigenetic toolkit of insects underwent rapid evolutionary changes, including strong differences in DNA methylation between hymenopteran species [82]. Importantly, the epigenetic toolkit has not been comprehensively compared between major taxa in either nematodes or hymenopterans. This, therefore, represents a major research endeavour that may result in exciting and important findings in the future. 

Finally, the control of insect polyphenisms by histone modifications is not restricted to ants; beetle horn development was also shown to be controlled through histone deacetylases [83]. Therefore, epigenetic reprogramming might represent a cornerstone in the regulation of polyphenisms in development, with an important role in histone modifications. However, more studies are needed to obtain a comprehensive picture of the type of modifications used to control polyphenisms. It is also currently unknown if genomic features, e.g., gene duplications of particular histone-modifying enzymes, precede the acquisition of epigenetic reprogramming of polyphenisms. For example, the epigenetic toolkit of *P. pacificus* and *C. elegans* shows substantial differences in the number of genes encoding histone-modifying enzymes [78]. Therefore, although it is challenging given their rapid evolution, studying the species specificity of the epigenetic toolkit of plastic animals may be a highly rewarding research direction. 

## 8. Microevolution: Natural Variation of Polyphenism Through *cis*-Regulatory Elements of *Pristionchus eud-1*

**Figure 3:**
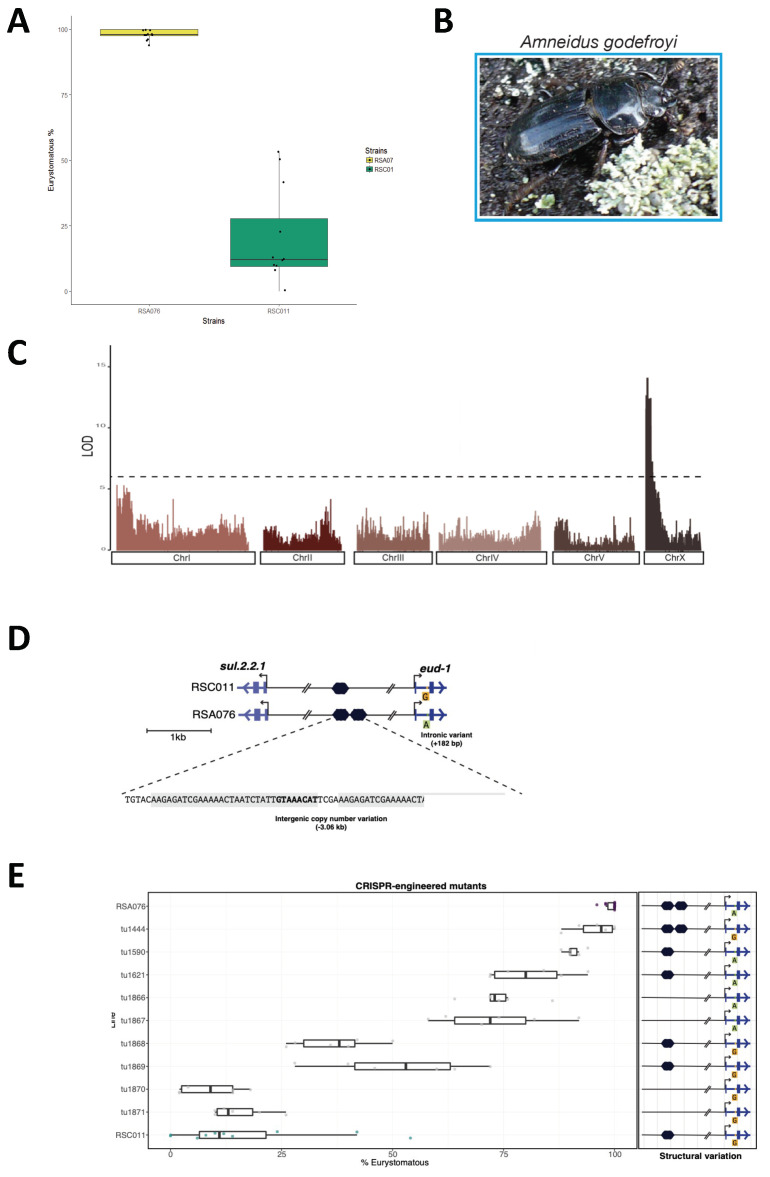
Natural variation and microevolution of mouth-form frequencies are caused by *cis*-regulatory mutants at the *eud-1* locus. (**A**) Two *P. pacificus* isolates from high-altitude locations on La Réunion Island show opposite mouth-form preferences (Eu % at the y-axis). While RSA076 is preferentially Eu under laboratory conditions, RSC011 is mostly St. (**B**) The stag beetle *A. godefroyi* is the host of high-altitude populations of *P. pacificus*. (**C**) A quantitative trait locus analysis of recombinant inbred lines of RSA076 and RSC011 reveals a single QTL, which is located at the *eud-1* locus, the major developmental switch gene of *P. pacificus* mouth-form plasticity. (**D**) *Cis*-regulatory differences in the *eud-1* locus between both isolates include copy number variations of a Forkhead binding site, a potential transcription factor regulating mouth-form polyphenism with RSA076 having two copies, whereas RSC011 has only a single copy of this binding site. An additional regulatory site is a single nucleotide switch in the first intron of *eud-1*. (**E**) CRISPR engineering was used the eliminate the binding sites and to swap the variant nucleotide in intron 1 in the original RSA076 background, resulting in increased St mouth-form frequencies. Note that in this panel, Eu frequencies are depicted at the x-axis with the two parental lines (RSA076 and RSC011) at the top and the bottom of the panel. Redrawn with permission from Dardiry et al., *PLoS Biology* 2023, *21*, e3002270.

Mechanistic studies in developmental genetics and epigenetics are usually disentangled from evolutionary investigations; therefore, the importance of elucidated molecular mechanisms for evolutionary diversification often remains unclear [4,84]. For example, the genetic and experimental identification of *eud-1* and H4K12ac modification at the *eud-1* locus for the control of the mouth-form polyphenism, as described above, do not provide any evidence on their own for the importance of these regulatory mechanisms for the evolution of feeding polyphenism. More generally, it is currently unknown whether natural variation in mouth-form plasticity is locally adapted and how genes in the mouth-form GRN might evolve at the micro- and macro-evolutionary levels. However, *P. pacificus* was originally set up as a model system in evolutionary developmental biology to specifically link mechanistic investigations with evolutionary biology [22,85]. The available collection of *P. pacificus* wild isolates as living cultures permits genetic and experimental studies that can link development and evolution in an interdisciplinary manner. Specifically, comparisons between wild isolates and closely related species can provide insight into the microevolutionary divergence of polyphenisms and associated mechanisms. Indeed, a recent investigation provided strong evidence for substantial natural variation of the feeding polyphenism and its adaptive value in *P. pacificus* (Figure 3). A collection of *P. pacificus* isolates from the stag beetle *Amneidus godefroy*, restricted to high altitude locations on La Réunion Island in the Indian Ocean, exhibited strong population differentiation with highly Eu and highly St strains (Figure 3A,B) [36]. Recombinant-inbred-line (RIL) and quantitative trait locus (QTL) analyses of one pair of a preferentially Eu and a preferentially St strain revealed a single QTL to control the natural variation in mouth-form preference (Figure 3C). This QTL controlling the phenotypic divergence of mouth-form polyphenism was subsequently shown to be at the *eud-1* locus (Figure 3D) [36]. Through CRISPR engineering experiments, several *cis*-regulatory elements in the promoter and the first intron of *eud-1* were shown to act synergistically to control the level of *eud-1* expression (Figure 3E). Thus, divergent combinations of *cis*-regulatory elements control the microevolution of polyphenisms.

## 9. Macroevolution: Conservation of *eud-1*-Regulated Switch in Other Diplogastrids

The identification of the sulfatase *eud-1* as a developmental switch in genetic and epigenetic studies, as well as a target of natural variation in closely related wild isolates of *P. pacificus*, highlights its importance in mouth-form regulation [36,51]. But what about macroevolution and the evolution of this switch over larger evolutionary distances? One possibility is that the *eud-1* switch is an anomaly that only recently evolved in *Pristionchus* and may not be relevant to understanding mechanisms of mouth-form plasticity in diplogastrid nematodes. To tackle such questions, a more ‘basal’ member of the Diplogastridae, *Allodiplogaster sudhausi* was recently established as a model for functional investigations, allowing for comparative approaches. *A. sudhausi* is similarly hermaphroditic, displays adult mouth-form plasticity, and, importantly, CRISPR engineering can be used to generate knock-out mutants through reverse genetics approaches [86]. Studying this nematode provides the opportunity to gain a broader context of *P. pacificus* mouth-form polyphenism and to link the underlying processes to the macroevolutionary level. Notably, *A. sudhausi* was shown to have undergone whole genome duplication, adding an increased level of complexity to the evaluation of polyphenic trait evolution [86]. 

Strikingly, the use of additional dietary conditions resulted in the identification of a new third mouth-form in *A. sudhausi* that is not known from *P. pacificus* (Figure 1E–G) [87]. *A. sudhausi* adults became St on *E. coli* bacteria, Eu after predating exclusively on *C. elegans* nematodes and formed a third mouth-form, termed Teratostomatous (Te), on *Penicillium camemberti* fungal diets as well as starved plates (Figure 1E–G). The finding of an evolved trimorphism from an ancestral dimorphism highlights the importance of mouth-form plasticity to enable morphological novelty and testifies to its significance for evolutionary divergence. This third mouth-form additionally displayed cannibalistic behaviour against genetically identical kin, indicating behavioural consequences of this particular polyphenism [87]. Note that the evolution of trimorphism from dimorphic ancestors is rare but known to occur in nematodes and beetles.

Two genes that are homologous to the *P. pacificus eud-1* sulfatase were identified in *A. sudhausi* due to the whole genome duplication. Notably, double mutant knock-outs in these genes led to an all-St phenotype under all diets and conditions [87]. This is the same phenotype as seen in *P. pacificus eud-1* mutants [51]. Therefore, the sulfatase genes maintain their role as a developmental switch in mouth-form regulation across vast evolutionary distances. There are two major takeaways: Firstly, the role of *eud-1* and its homologs are likely conserved within the Diploagstridae family, and secondly, the alternative polyphenism enabled the evolution of a third mouth-form, which also appears to be under the control of the highly conserved *eud-1*/sulfatase gene [87].

## 10. Evolution of a Switch Through a Single Locus: Striking Similarities Between *Pristionchus eud-1* and *Arabidopsis FLC*

The re-identification of *eud-1* in independent genetic, epigenetic, natural variation, and macroevolutionary studies in *P. pacificus* and other nematodes is striking but not unprecedented. The work of Caroline Dean and others on the control of flowering time in *Arabidopsis thaliana* shows many parallels with the major role of *flowering locus C* (*FLC*) (for an extended review see, [88]). In general, plants have to couple their development and reproduction to favourable environmental conditions, which requires the sensing and integration of cues that properly indicate seasonal changes. There is substantial natural variation with regard to flowering time: many wild *A. thaliana* accessions flower rapidly with many generations being produced within one summer, so-called ‘summer annuals’, whereas other accessions require over-wintering and only flower after long exposure to the cold, a process referred to as ‘vernalisation’. Floral transition is the major developmental switch in plants and has been genetically investigated in great detail. In *A. thaliana*, the *FLC* locus represents the central component in the regulation of this switch and is encoded by a MADS box transcription factor [89]. In late flowering ecotypes, vernalisation is controlled by *FLC*, which is expressed in the new seedling and prevents flowering. Exposure to prolonged cold temperatures indicating winter will repress *FLC* and, together with parallel pathways, will result in flowering during springtime. Similar to *P. pacificus eud-1*, *A. thaliana FLC* is also epigenetically regulated and subject to extensive natural variation. Epigenetic silencing of *FLC* involves histone methylation at *FLC* as well as a Polycomb-like mechanism [88]. These original observations have recently been integrated with theoretical modelling, which suggests multiple thermosensitive inputs [90,91]. Finally, ecotypes of *A. thaliana* have different alleles for the *FLC* gene that correspond to early and late flowering. Thus, similar to *eud-1*, *FLC* is extensively regulated through epigenetic modifications and transcriptional control and harbours substantial natural variation. These features are likely general principles underlying polyphenisms and developmental switching beyond *A. thaliana* and *P. pacificus*.

## 11. Conclusions

In conclusion, recent advances in understanding the regulation of polyphenisms have led to two major insights. First, polyphenisms require underlying genetic programs similar to hard-wired developmental processes and second, epigenetic reprogramming represents the key feature for integrating environmental variation into developmental programs with robust organismal readouts. These findings are of importance to developmental and evolutionary biologists alike; however, the findings have contrasting implications. Evolutionary biologists should start to acknowledge the significance of phenotypic plasticity for evolution (see Futuyma vs. West-Eberhard in 5). In particular, phylogenetic studies in vertebrates, insects, and nematodes suggest a strong link between the origins of novelty and plasticity [30,92,93]. Together with the growing understanding of its molecular regulation, this body of evidence points towards the powerful role of polyphenisms as major drivers of evolutionary novelty. Alas, despite ever-growing evidence for its importance, resistance against this idea remains. 

For developmental biologists, polyphenisms might become important study systems for other reasons. For instance, the binary readout of alternative phenotypes is ideal for representing simple model systems to investigate how environmental variation influences developmental processes. Importantly, this could also go beyond studies of normal ontogenesis and include developmental malformation during cancer. Recent studies of human colorectal cancer provided strong evidence for transcriptional phenotypic plasticity in tumours, as intra-tumoral ancestry is a poor predictor of gene expression and sub-clonal evolution [94]. Indeed, a recent review pointed towards epigenetics as a major mediator of plasticity in cancer and highlighted that three mechanisms shape the major features of cancer and tumour cells: genetics, environment, and stochasticity [95]. These same three features are also the prominent regulators of mouth-form polyphenism in the nematode *P. pacificus*. As mentioned above, the *P. pacificus* feeding polyphenism is a bistable switch incorporating genetic and environmental cues that also show elements of stochasticity [37]. The simple alternative readout and the ease with which some polyphenisms can be studied under laboratory conditions might make them a powerful study system beyond plasticity itself. Thus, polyphenisms can provide important insight into the Nature vs. Nurture debate and help towards a better integration of genetic and environmental influences in health and disease.

## Data Availability

The authors confirm that there is no data to be made available for this review.
